# Identification of Nuclear Genetic Loci Linked to Clinical Features of the m.3243A>G Mitochondrial DNA Variant

**DOI:** 10.1212/NXG.0000000000200411

**Published:** 2026-07-16

**Authors:** Róisín M. Boggan, Theodora-Dafni Michalettou, Yi Shiau Ng, Imogen G. Franklin, Lucas T. Cortés, Charlotte L. Alston, Emma L. Blakely, Boriana Büchner, Enrico Bugiardini, Kevin Colclough, Catherine Feeney, Michael G. Hanna, Andrew T. Hattersley, Thomas Klopstock, Cornelia Kornblum, Michelangelo Mancuso, Kashyap A. Patel, Robert D.S. Pitceathly, Chiara Pizzamiglio, Holger Prokisch, Jochen Schäfer, Andrew M. Schaefer, Maggie H. Shepherd, Annemarie Thaele, Rhys H. Thomas, Doug M. Turnbull, Cathy E. Woodward, Robert McFarland, Robert W. Taylor, Heather J. Cordell, Sarah J. Pickett

**Affiliations:** 1 Biosciences Institute, Faculty of Medical Sciences, Newcastle University, United Kingdom;; 2 Mitochondrial Research Group, Faculty of Medical Sciences, Newcastle University, United Kingdom;; 3 Translational and Clinical Research Institute, Faculty of Medical Sciences, Newcastle University, United Kingdom;; 4 Directorate of Neurosciences, Royal Victoria Infirmary, Newcastle upon Tyne Hospitals NHS Foundation Trust, United Kingdom;; 5 NHS Highly Specialised Services for Rare Mitochondrial Disorders, Newcastle upon Tyne Hospitals NHS Foundation Trust, United Kingdom;; 6 Department of Neurology, Friedrich-Baur-Institut, University Hospital of the Ludwig-Maximilians-University (LMU Klinikum), Munich, Germany;; 7 Department of Neuromuscular Diseases, University College London Queen Square Institute of Neurology, London, United Kingdom;; 8 NHS Highly Specialised Service for Rare Mitochondrial Disorders, Queen Square Centre for Neuromuscular Diseases, The National Hospital for Neurology and Neurosurgery, London, United Kingdom;; 9 Exeter Genomics Laboratory, Royal Devon University Healthcare NHS Foundation Trust, Exeter, United Kingdom;; 10 Institute of Biomedical and Clinical Science, University of Exeter Medical School, United Kingdom;; 11 Munich Cluster for Systems Neurology (SyNergy), Germany;; 12 German Center for Neurodegenerative Diseases (DZNE), Munich, Germany;; 13 Center for Neurology, Department of Neuromuscular Diseases, University Hospital Bonn, Germany;; 14 Department of Clinical and Experimental Medicine, Neurological Institute, University of Pisa, Italy;; 15 Institute of Human Genetics, School of Medicine, Technische Universität München, Germany;; 16 Institute of Neurogenomics, Helmholtz Zentrum München - German Research Center for Environmental Health, Neuherberg, Germany;; 17 German Center for Child and Adolescent Health (DZKJ), Munich, Germany;; 18 Department of Neurology, Universitätsklinikum Carl Gustav Carus, Technische Universität Dresden, Germany;; 19 Royal Devon University Healthcare NHS Foundation Trust, Exeter, United Kingdom;; 20 NIHR Biomedical Research Centre, University of Exeter Medical School, United Kingdom;; 21 Department of Neurology, Martin-Luther-University Halle-Wittenberg, Halle (Saale), Germany;; 22 Neurogenetics Unit, The National Hospital for Neurology and Neurosurgery, London, United Kingdom; and; 23 Population Health Sciences Institute, Faculty of Medical Sciences, Newcastle University, United Kingdom.

## Abstract

**Background and Objectives:**

Mitochondrial DNA (mtDNA) disorders exhibit striking clinical variability that is poorly explained by known factors such as variant heteroplasmy, age, or sex. Nuclear genetic modifiers likely play a significant role in this heterogeneity. We aimed to characterize the nature of nuclear genetic involvement for 2 common syndromic presentations of the common pathogenic mtDNA variant, m.3243A>G: mitochondrial encephalomyopathy, lactic acidosis, and stroke-like episodes (MELAS) and maternally inherited diabetes and deafness (MIDD).

**Methods:**

We assembled a multicenter cohort of clinically ascertained carriers of m.3243A>G (total n = 488), identifying 198 individuals across 76 pedigrees suitable for genetic linkage analysis. We investigated 4 clinical features characteristic of MELAS and MIDD: diabetes, hearing impairment, stroke-like episodes, and encephalopathy. Haseman-Elston regression-based genetic linkage analysis was performed to identify regions of the nuclear genome cosegregating with these features. The effects of m.3243A>G heteroplasmy, age, and sex were accounted for using logistic regression; empirical significance thresholds were determined through feature-specific gene-dropping simulations. Association analyses were performed in 247 individuals using single-variant (SAIGE) and gene-based approaches (SAIGE-GENE+ and MAGMA) to refine candidate loci within a significant linkage region.

**Results:**

We identified significant genetic linkage to encephalopathy (chromosome 7q22; LOD = 3.72), and regions suggestive of genetic linkage on chromosomes 1, 5, 6, 11, and 13, for encephalopathy and stroke-like episodes. No linkage was identified for diabetes or hearing impairment. Association analysis within the chromosome 7 region identified variant rs62500792 (intergenic between *SDHAF3* and *TAC1*) with the lowest *p* value (3.7 × 10^−5^), yet no variants reached the proportional significance threshold (5.3 × 10^−6^). Gene-based analyses highlighted *PLOD3* (*p* = 3.9 × 10^−3^) and *IMMP2L* (*p* = 6.4 × 10^−3^) as candidates, as each showed the strongest gene-level signals within the linkage region across complementary burden-testing methods, although neither reached corrected significance thresholds.

**Discussion:**

The nuclear genetic architecture modifying m.3243A>G differs across clinical features. Severe neurologic features (encephalopathy and stroke-like episodes) may be influenced by a small number of nuclear genes with relatively large effect sizes, whereas the nuclear contribution to diabetes and hearing impairment appears more polygenic. This study highlights the value of large, well-characterized patient cohorts in identifying modifier loci and advancing knowledge of the mechanisms underlying phenotypic variability in mtDNA disease.

## Introduction

Maternally inherited mitochondrial DNA (mtDNA) disorders affect ∼1 in 5,000 individuals, can arise at any age, are often multisystemic, and are extremely clinically variable.^1^ The cause of this extreme heterogeneity, even within groups of individuals carrying the same pathogenic mtDNA variant, is poorly understood, making disease diagnosis and prognosis exceptionally challenging. This is exemplified by disease associated with the most common pathogenic mtDNA variant, m.3243A>G (RefSeq:NC_012920.1), within *MT-TL1*. Although initially identified as a cause of mitochondrial encephalomyopathy, lactic acidosis, and stroke-like episodes (MELAS),^2^ this syndrome affects less than 20% of all m.3243A>G disease patients.^3^ Of interest, approximately 40% of m.3243A>G carriers present with diabetes mellitus and hearing impairment, often observed together and characterized as maternally inherited diabetes and deafness (MIDD).^3,4^ However, affected individuals can present with a broad range of clinical features, including severe CNS presentations such seizures and cerebellar ataxia and features outside the CNS such as skeletal myopathy, cardiomyopathy, and renal impairment. Estimates of the carrier frequency of m.3243A>G are 40–70 times higher than disease prevalence, suggesting that carriers are clinically asymptomatic or undiagnosed.^5,6^

A contributing factor to mtDNA disease complexity is the co-existence of mtDNA molecules carrying both reference and alternative alleles within the same cell (known as heteroplasmy); m.3243A>G levels can vary between individuals, tissues, and cells.^7,8^ Variation in m.3243A>G heteroplasmy within commonly tested tissues and patient age both contribute to phenotypic variation, but the majority (∼70–80%) remains unexplained.^7-11^ Moreover, patients with similar m.3243A>G heteroplasmy can present with very different phenotypes and disease severity, highlighting how difficult it is to provide patients with satisfactory genetic counselling.

The mitochondrial genome encodes 13 protein-coding genes, 2 rRNAs, and 22 tRNAs, while the nuclear genome encodes ∼1,150 proteins required for mitochondrial function^12^; pathogenic variants in either genome can result in mitochondrial disease. We have previously demonstrated that nuclear genetic variation contributes to the phenotypic diversity of m.3243A>G-related disease; moderate to high heritability estimates indicate that additive genetic factors play a much larger role in determining disease phenotype than m.3243A>G proportion, age, and sex combined.^11^ However, these factors may be monogenic or polygenic, and the specific factors have yet to be identified.

To understand the nuclear genetics contributing to this heritability, we assembled a unique multicenter cohort of well-characterized patients and their maternal relatives, comprising 76 pedigrees. This enabled mapping of the nuclear genetic landscape associated with m.3243A>G disease phenotypes using nonparametric genetic linkage analysis. This technique has been highly successful in identifying regions of the nuclear genome that co-segregate with numerous disease phenotypes (such as type 1 diabetes,^13^ Crohn disease^14^) and has the advantage of being able to detect effects due to rare and common variants, along with family-specific effects.^15^ We considered 4 m.3243A>G disease-related clinical features that define MELAS and MIDD: encephalopathy, stroke-like episodes, diabetes mellitus, and hearing impairment. We accounted for known risk factors by using the residuals from logistic regression, identifying regions of the nuclear genome most likely to contain disease modifying variants.

## Methods

### Study Cohort

Our combined cohort consists of 488 individuals from 5 clinical centers (eTable 1; 192 male; median age = 41.7, IQR = 22.9), all genetically confirmed to carry the m.3243A>G variant (median m.3243A>G heteroplasmy 0.743, IQR = 0.053, range = 0.002–1.000).

### Standard Protocol Approvals, Registrations, and Patient Consents

Written informed consent from all individuals was obtained before study inclusion, and all clinical investigations were evaluated according to the Declaration of Helsinki.

Participants were recruited from multiple centers contributing to UK and European Mitochondrial Disease Patient Cohorts. Individuals from the Newcastle component of the Mitochondrial Disease Patient Cohort, the United Kingdom, were reviewed and investigated by the NHS Highly Specialized Service for Rare Mitochondrial Disorders of Adults and Children in Newcastle upon Tyne. Ethical approval was granted by the Newcastle and North Tyneside Research Ethics Committee (13/NE/0326). Mitochondrial disease patient tissue was provided by the Newcastle Mitochondrial Research Biobank (16/NE/0267).

Additional participants were included from the University College London (UCL) arm of the Mitochondrial Disease Patient Cohort, the United Kingdom, covered under the same overarching approval (13/NE/0326); with site-specific ethical approval provided by the Queen Square Research Ethics Committee, London, the United Kingdom (09/H0716/76).

Data and samples were also obtained from the German network for mitochondrial disorders “mitoNET.” The clinical Registry (mitoREGISTRY) was approved by the Ethics Committee of the LMU Munich (182-09) and local Ethic Committees of all contributing medical institutions. The Biobank (mitoSAMPLE) was approved by the Ethics Committee of the Technical University Munich (200/15 S-SR) and local Ethic Committees of all contributing medical institutions.

Further participants were identified through routine clinical referrals to the Exeter Genomics Laboratory at the Royal Devon and Exeter Hospital for diagnostic genetic screening related to diabetes mellitus and were shown to have m.3243A>G-related disease. The study was approved by the North Wales ethics committee (17/WA/0327). As referral was primarily for diagnostic testing related to diabetes mellitus, systematic phenotyping for other clinical features was limited; diabetes status was available for most individuals, whereas data for other clinical features were available for a smaller subset (eTable 1). Participants were also included from the University of Pisa, enrolled in the “Nationwide Italian Collaborative Network of Mitochondrial Diseases.” The database establishment (and its use for scientific purposes) was approved by the local Ethical Committees of the single centers.

### mtDNA Molecular Analysis

m.3243A>G heteroplasmy was quantified within DNA samples that had been extracted from whole blood, urine or muscle samples by pyrosequencing on the Pyromark Q24 platform, as previously described and validated (test sensitivity >3% mutant mtDNA).^16^ Blood levels of m.3243A>G were corrected using the previously published formula.^8^ Where multiple blood m.3243A>G heteroplasmy measurements were available, the mean age-adjusted blood level was used. Where blood m.3243A>G heteroplasmy was not available, urine m.3243A>G heteroplasmy was used (n = 18), correcting for sex using previously published formulas.^8^ Where neither blood nor urine measures were available, we used assessments from skeletal muscle tissue (n = 5); no correction was performed on measures from muscle tissue, as variant heteroplasmy is known to remain stable over time.^8^

### Clinical Assessments

Assessments of clinical features in the form of Newcastle Mitochondrial Disease Adult Scale (NMDAS) scores were available within the cohorts from Newcastle, Germany, and Italy. The NMDAS is a clinically validated scale used to assess patients with mitochondrial disease, consisting of 29 questions rated on an ordinal scale of 0–5 (0 = unaffected, 5 = severely affected).^17^ We considered 4 questions from the NMDAS, covering stroke-like episodes, encephalopathy, diabetes, and hearing (eTable 2). In-line with previous studies,^11^ where multiple clinical assessments were available for an individual, we used the maximum NMDAS score and the youngest age at which that score was attained (or the age at most recent assessment if NMDAS = 0). We converted these semiquantitative scores into binary assessments of clinical feature affection status (0 = unaffected/asymptomatic, 1 = affected) using previously published thresholds, developed with clinical guidance (Table 1).^11^ For the cohorts from UCL and Exeter, full NMDAS assessments were unavailable, and therefore, a clinician used these descriptors to assign a binary score for each clinical feature, using information available within medical notes. The median age at last assessment was 41.7 (IQR = 22.9), and the median corrected m.3243A>G heteroplasmy was 0.743 (IQR = 0.053); individual cohort demographics are summarized in eTable 1.

**Table 1:** Study Cohort

Clinical feature	Definition	NMDAS threshold score	Phenotyped cohort	Linkage cohort		Association cohort
			Total (affected)	Total (affected)	Number of informative pedigrees	Total (affected)
Encephalopathy	At least history of encephalopathy	1	378 (80)	156 (27)	61	247 (49)
Stroke-like episodes	At least transient focal sensory symptoms	1	378 (82)	156 (24)	61	—
Hearing impairment	At least moderate deafness (e.g., regularly requiring repetition), not fully corrected with hearing aid)	3	392 (218)	162 (79)	62	—
Diabetes mellitus	At least impaired glucose tolerance (in absence of intercurrent illness)	2	487 (274)	198 (93)	76	—

Abbreviation: NMDAS = Newcastle Mitochondrial Disease Adult Scale; SNP = single nucleotide polymorphism.

Phenotyped cohort: cohort used for logistic regression analyses to account for the effect of established risk factors. Linkage cohort: individuals within pedigrees with both SNP genotype and phenotype information available. Association cohort: all individuals with SNP genotype, phenotype information and covariate data available. The linkage and association cohorts are smaller than the phenotyped cohort as genotype information was not available for all individuals and not all individuals were members of pedigrees.

### Accounting for Established Influences of m.3243A>G-related Disease

To determine the effects of age, m.3243A>G heteroplasmy, and biological sex on the development of each clinical feature, we performed a logistic regression in R (version 3.6.0) using the “glm()” function with the binomial option in the “stats” package. Odds ratios were calculated from the exponentials of the coefficients estimated by this logistic regression model; they represent the odds that an individual will move from unaffected to affected given an increase in age (decade) or 10% m.3243A>G variant level. McFadden's pseudo-*R*^2^ values were calculated using the following formula: 1-log(L_c_)/log(L_null_), where L_c_ is the maximum likelihood of the fitted model and L_null_ is the maximum likelihood of the null. To account for the effects of these known risk factors in downstream linkage analysis, model residuals were extracted and used as a proxy phenotype.

### SNP Genotyping, Quality Control, and Imputation

DNA samples from 488 individuals were single nucleotide polymorphism (SNP) genotyped using the UKB WCSG array, known as the UK Biobank Axiom® Array, designed by the UK Biobank Array Design Group and performed by ThermoFisher. Initial data quality control was performed in Axiom® Analysis Suite (AxAS: version 4.0.3.3) using internal Axiom® quality metrics (Fisher's Linear Discriminant, FLD ≥4; Call rate ≥97; Heterozygous ratio offset ≥0; Homozygous ratio offset ≥0). Data for 645,115 SNPs were subsequently exported in PLINK format.

**Figure 1:**
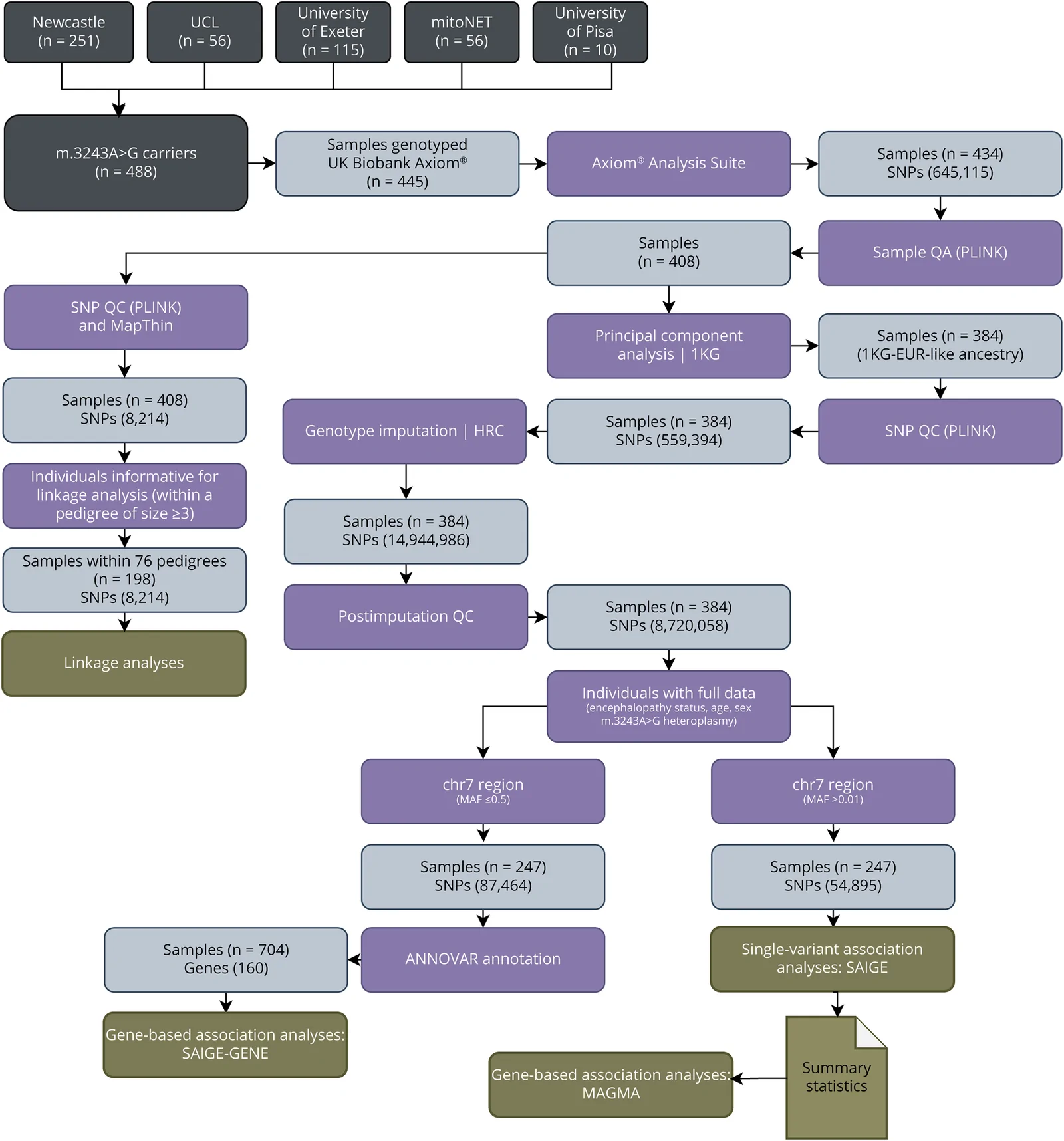
Flowchart Summarizing Cohort and SNP Quality Control

A second round of quality control (with SNP exclusion thresholds chosen to be specific for linkage analysis) was performed in PLINK (version 1.90b6.6)^18^ in accordance with recommendations,^19^ and visualization was performed in R (version 3.6.0). Nuclear SNPs were pruned by minor allele frequency (>0.35) and linkage disequilibrium (using the PLINK command “--indep 50 5 2”). SNPs were subsequently thinned, by selecting the 2 SNPs with the highest heterozygosity in each 2.4 cM window using the program MapThin,^20^ resulting in a final set of 8,214 autosomal SNPs for linkage analysis. 408 genotyped samples remained after the completion of quality control procedures (Figure 1).

Before performing the association analyses, we used nuclear genetic principal component analysis (PCA) to compare the structure of our cohort with data from the 1000 Genomes Project (1 KG; containing sequences from individuals from 5 genetically distinct “super populations”; AFR, EUR, EAS, AMR, and SAS).^21^ Eigenvectors and eigenvalues were calculated in PLINK (version 1.90b7.2) and indicated that the first 2 principal components (PCs) explained 76% of genotypic variance, while the third and fourth PCs accounted for only 13% of the variance (eFigure 1A). The first 2 PCs were plotted to visualize genetic ancestry clusters (eFigure 1B). Most individuals in our cohort clustered with individuals of 1000 Genomes Project European-like (1KG-EUR-like) genetic ancestry. Twenty-four individuals who fell outside of this cluster were removed from downstream association analyses to reduce the effects of population stratification.

To prepare genotype data for imputation before association analysis, all genotyped SNPs that passed initial quality control were filtered by minor allele frequency (>0.01), Hardy-Weinburg Equilibrium (HWE *p* > 10^−8^) and missingness (<0.03), and ambiguous (A:T and C:G) SNPs were removed, to produce a set of 559,394 biallelic SNPs. Imputation was performed using the Michigan Imputation Server^22^ and the Haplotype Reference Consortium (version r1.1.2016) reference panel, which consists of 64,940 haplotypes of primarily European ancestry. Postimputation, 8,720,058 SNPs with imputation quality *R*^2^ > 0.8 and HWE *p* > 10^−8^ were taken forward for downstream association analyses. Association analyses were performed on a subset of these variants, located in the identified region of significant genetic linkage on chromosome 7 (Figure 1).

### Linkage Analysis

A variant of Haseman-Elston regression-based linkage analysis, which regresses estimated identity-by-descent (IBD) between relative pairs on the squared sums and squared differences of trait values of the pairs, was used. This approach is relatively robust to trait non-normality. Population parameters (mean, variance, heritability) were derived from the full sample. Multipoint logarithm of the odds (LOD) scores for the presence of a quantitative trait locus (QTL) were estimated by Merlin-REGRESS, as implemented in Merlin (v1.1.2).^23^ To account for known risk factors, we used the residuals from logistic regression models (regressing the trait on the risk factors) as proxy-phenotypes.

### Empirical Significance Thresholds

To determine the ability of the data to detect significant regions of genetic linkage, we performed 1,000 gene-dropping simulations in Merlin-REGRESS using the “--simulate” option, retaining the allele frequencies, marker positions, and missing genotype patterns of the data. Simulated data were analyzed in an identical way to the actual data. Regions of suggestive and significant linkage were initially identified using the widely accepted LOD score thresholds of LOD ≥1.86 and LOD ≥3.3, respectively.^24^ Linkage peaks were defined using a base pair (bp) window surrounding each maximum LOD score. The performance of several window sizes was tested against manual identification of linkage peaks (by visual inspection) within the results data; ±15 Mb resulted in the best concordance with manual identification of linkage regions.

**Figure 2:**
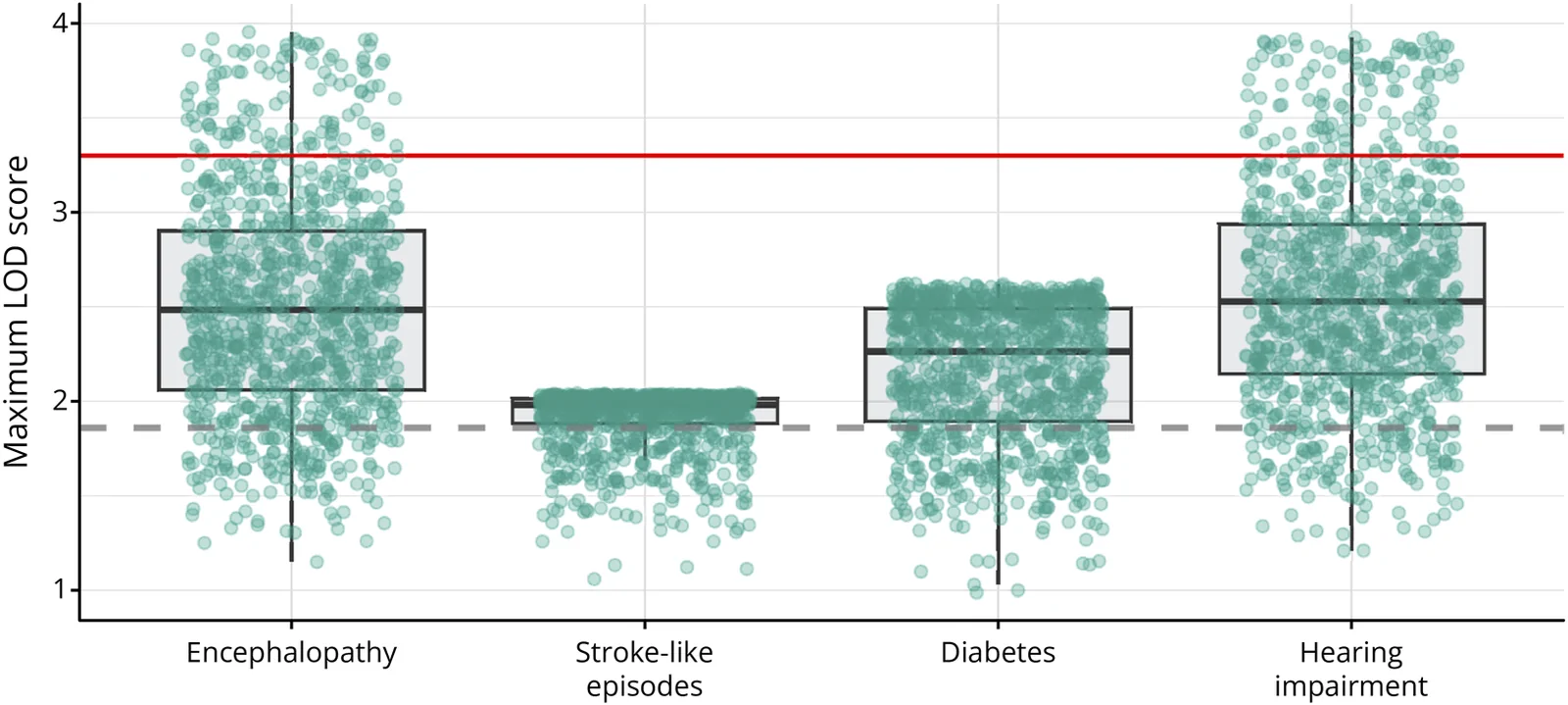
Maximum LOD Scores Achieved Through Simulation of Genetic Data Each point represents the maximum LOD score per simulated genome scan, per clinical feature. Nominal thresholds for significant (LOD ≥3.3; horizontal red line) and suggestive (LOD ≥1.86; horizontal dashed gray line) linkage are shown for reference 24. Box plots depict the median, 25th, and 75th quantiles and whiskers extend from the hinge to the largest/smallest values no further than 1.5 * IQR from the hinge. LOD = logarithm of the odds.

The distribution of simulated LOD scores under the null model differed considerably between clinical features so applying the standard significance threshold of LOD ≥3.3 was not appropriate (Figure 2).^24^ Therefore, autosomal genome-wide empirical suggestive and significant LOD score thresholds for each feature were derived from the simulated data results (eTable 3). “Suggestive of genetic linkage” was defined as ≥ the LOD score which appeared once per genome scan, and “significant genetic linkage” as ≥ LOD score which appeared 0.05 times per genome scan (i.e., once in 20 genome scans).

### Association Analyses

Associations with encephalopathy were tested using generalized mixed models on genotype dosages where we controlled for the effects of age, sex, and m.3243A>G heteroplasmy using data from 247 individuals of 1KG-EUR-like genetic ancestry (49 cases; 198 controls) with encephalopathy data and full covariate data (Figure 1). A sparse genetic relationship matrix (GRM) was computed using 165,979 hard-called SNP genotypes (minor allele frequency [MAF] >0.05; pruned for linkage disequilibrium) to further account for sample-relatedness and population structure. Single variant associations were tested using models implemented in SAIGE (v1.3.0),^25^ which performs saddle-point approximation to account for sample relatedness and imbalances in case-control numbers. Associations between 54,895 SNPs filtered for MAF >0.01 in the chromosome 7 region and encephalopathy status (binary; eTable 3) were tested. Functional annotation of imputed genotype data for the downstream gene burden analysis was performed using ANNOVAR (version: 2025-03-02).^26^ Gene burden analysis was performed using SAIGE GENE+.^27^ To capture as much variation as possible across all samples, we included loss of function, missense, and synonymous variants with MAFs ≤0.5 (87,464 SNPs). We visually inspected QQ-plots and calculated genome-wide genomic inflation factors (1.006 and 0.944 for GWAS and gene burden tests, respectively), which indicated that our models adequately accounted for population stratification due to sample relatedness.^28^ Using the summary-level statistics from the single variant association analysis and the EUR subset of 1 KG as reference to estimate Linkage Disequilibrium (LD), we also performed a gene-based analysis with MAGMA v1.08^29^ through FUMA.^30^ While SAIGE-GENE accounts for LD implicitly through the GRM, MAGMA controls for LD by using a PC regression model, better suited for detecting effects involving multiple markers with weak individual associations.

### Data Availability

Code for data analysis is available on GitHub.^31^

## Results

### m.3243A>G Heteroplasmy and Age Do Not Explain Clinical Variability

We assembled a study cohort of 488 individuals with m.3243A>G-associated disease recruited across multiple centers (Figure 1). This included 251 individuals from the Newcastle component of the UK Mitochondrial Disease Patient Cohort, 56 individuals from the UCL arm of the same cohort, 56 individuals from the German mitochondrial disease network mitoNET, 115 individuals identified through diagnostic referrals to the Exeter Genomics Laboratory, and 10 individuals from the Nationwide Italian Collaborative Network of Mitochondrial Diseases. Six individuals were represented in more than one contributing cohort; their clinical feature data were merged and counted once.

Previously, we established that m.3243A > G heteroplasmy (within commonly tested tissues) and age account for less than 20% of the variation in severity of m.3243A>G-related clinical features. We replicated this finding in our expanded cohort using binary feature data (eFigure 2), confirming that the proportion of variance explained is <20% for encephalopathy, stroke-like episodes, diabetes, and hearing impairment. We also observed a sex effect in the analysis of encephalopathy (OR_male_ = 1.70, CI = 1.01–2.85, *p =* 0.004). With the addition of biological sex as an explanatory variable, the proportion of observed phenotypic variation that can be explained in encephalopathy is still <10%.

### Genetic Linkage Identified in Neurologic Features of m.3243A>G-Related Disease

As these factors do not account for most of the clinical variation and nuclear modifiers are likely to contribute to this heterogeneity, we performed linkage analysis using the residuals from logistic regression as a quantitative phenotype and determined significance using feature-specific empirical suggestive and significant linkage LOD score boundaries. Pedigree-tracing within the cohort identified 198 individuals across 76 pedigrees, each comprising 3 or more informative members, who were eligible for linage analysis (Table 1, Figure 1).

**Figure 3:**
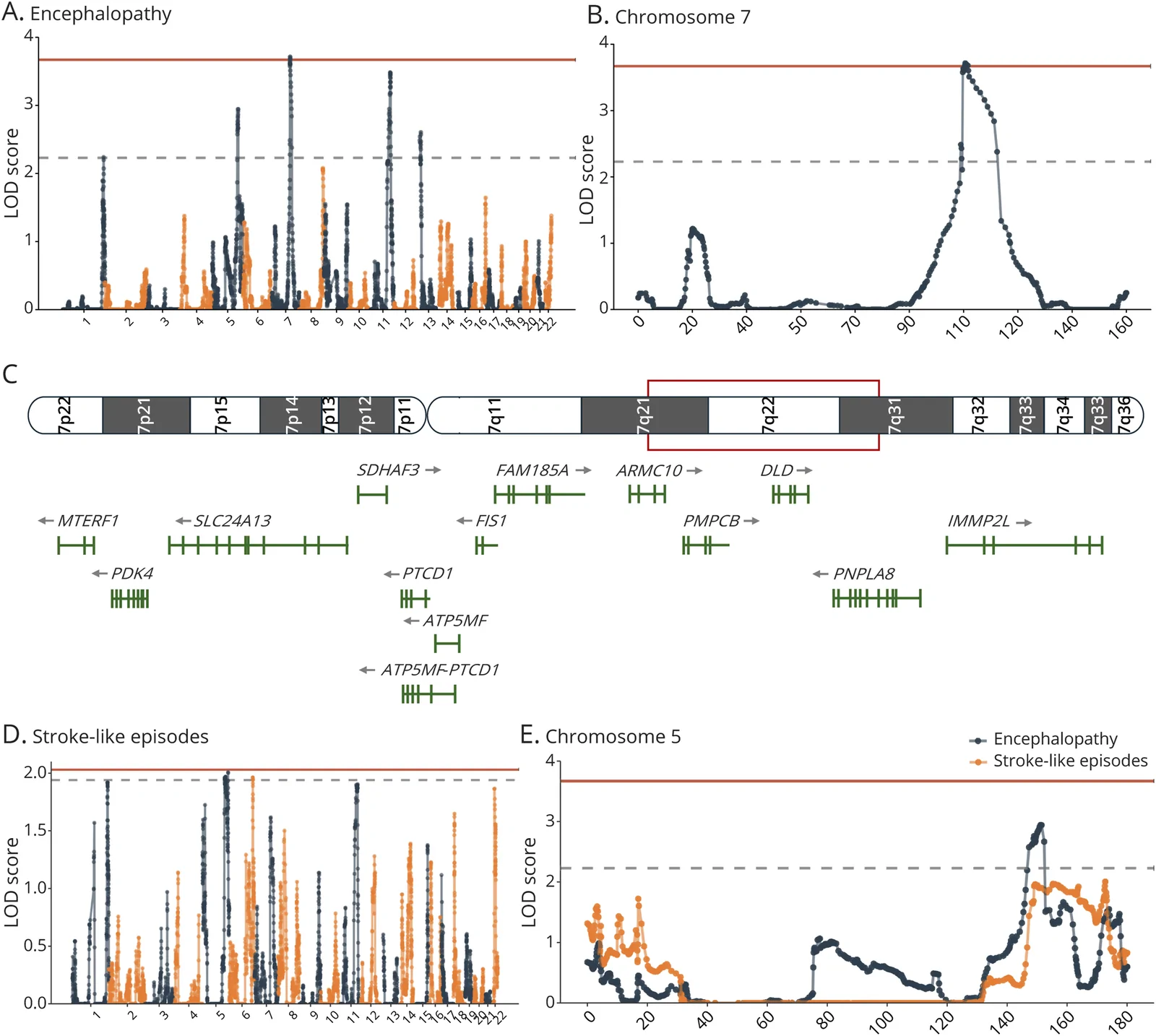
Linkage Results for Encephalopathy and Comparison With Results From Stroke-Like Episodes (A). Genome-wide linkage analysis of encephalopathy, empirical significance threshold LOD ≥3.67 (horizontal red line); empirical suggestive threshold LOD ≥2.23 (horizontal gray dashed line). A region on chromosome 7 (7q21.13–31.32) was identified as significant (LOD_max_ = 3.716) and a further 4 linkage regions were identified as suggestive of genetic linkage. (B). Encephalopathy chromosome 7 linkage; x-axis shows distance along chromosome in Mbp. (C). Identified genes in the highlighted region on chromosome 7 have a likely mitochondrial function/localization according to MitoCarta3.0^12^, positions and lengths of genes are relative, not to scale. (D). Stroke-like episodes, as recorded through the NMDAS assessment. Empirical suggestive threshold LOD ≥1.94, empirical significance threshold LOD ≥2.03. Two regions suggestive of genetic linkage were identified on chromosomes 5 (LOD_max_ = 2.01) and 6 (LOD_max_ = 1.96). (E) Comparison of chromosome 5 suggestive linkage regions for encephalopathy (LOD_max_ = 2.94), and stroke-like episodes (LOD_max_ = 2.01), stroke-like episodes LOD significance threshold (LOD ≥2.03) shown by red horizontal line; x-axis shows distance along chromosome in Mbp. LOD = logarithm of the odds.

Two of the most severe neurologic features associated with the m.3243A>G variant are stroke-like episodes and encephalopathy. For encephalopathy, we identified regions suggestive of genetic linkage on chromosomes 1 (LOD_max_ = 2.24), 5 (LOD_max_ = 2.94), 11 (LOD_max_ = 3.48), and 13 (LOD_max_ = 2.61; Figure 3A), and a region of significant genetic linkage on chromosome 7 (LOD_max_ = 3.72; 7q21.13–31.32; Figure 3B; eTable 3). There are >200 genes within this region, 14 of which have a likely mitochondrial function as identified by *MitoCarta3.0* (Figure 3C).^12^

For stroke-like episodes, we identified 2 regions suggestive of genetic linkage on chromosome 5 (LOD_max_ = 2.01) and chromosome 6 (LOD_max_ = 1.96; Figure3D); no regions reached our empirical significance threshold of 2.03, although the pattern of LOD scores under the null suggests that our cohort has limited power to detect large LOD scores. As expected, there is some correlation between encephalopathy and stroke-like episodes, both cardinal features of MELAS, (r∼0.54, *p* < 0.0001) and the region identified for stroke-like episodes on chromosome 5 (apex at 5q33.2) overlaps with a region showing suggestive evidence of linkage to encephalopathy (apex at 5q33.1; Figure 3E); this may indicate a shared nuclear factor that influences both clinical features.

### Non-neurologic Features of m.3243A>G-Related Disease Demonstrate a Polygenic Nature

**Figure 4:**
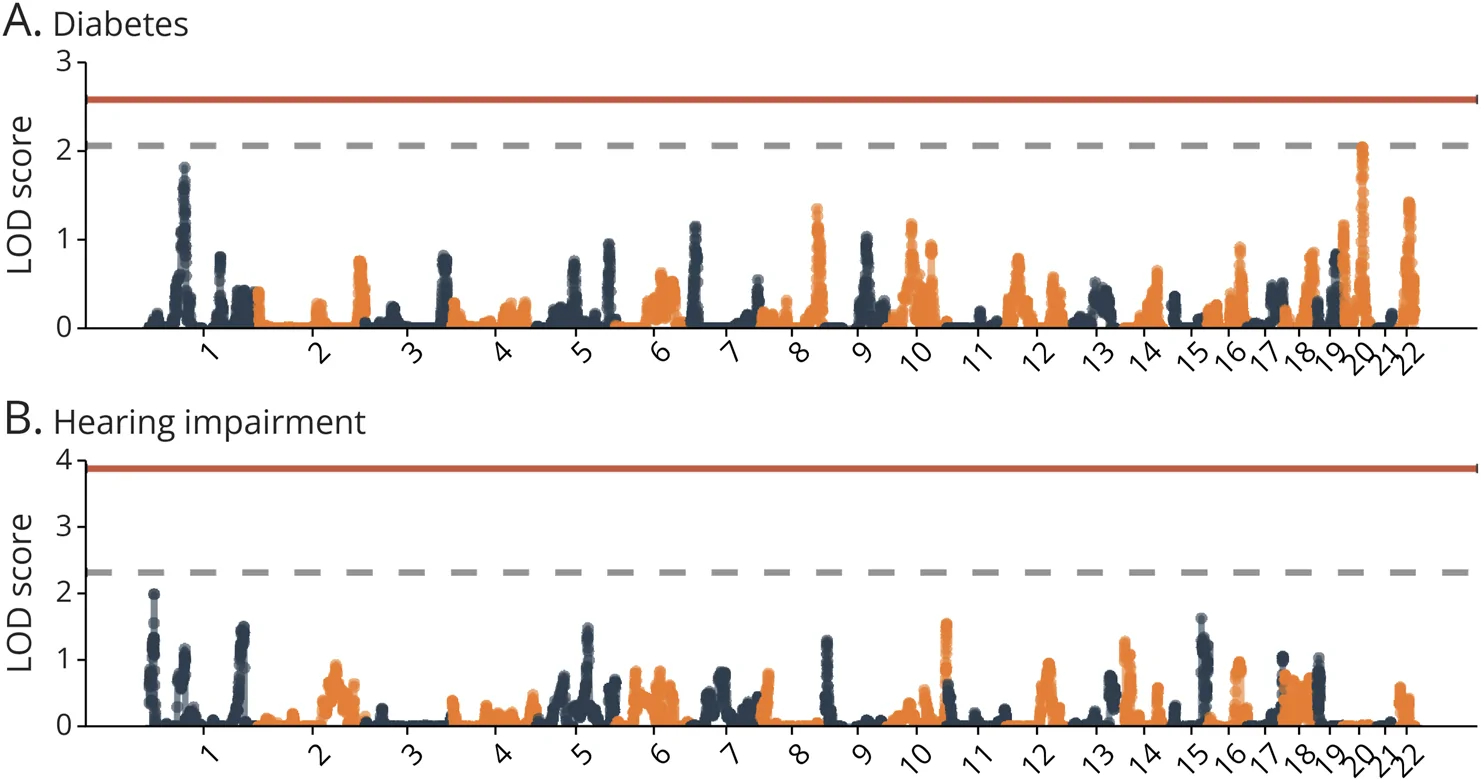
Linkage Results for Diabetes Mellitus and Hearing Impairment Using empirical LOD score thresholds there were no regions of suggestive or significant genetic linkage identified in either (A). m.3243A>G-related diabetes (empirical thresholds LOD ≥2.06, LOD ≥2.53 respectively) or (B). hearing impairment (empirical thresholds LOD ≥2.32, LOD ≥3.88). Empirical suggestive threshold depicted by dashed gray line; empirical significant threshold depicted by horizontal red line. LOD = logarithm of the odds.

We next investigated clinical features associated with maternally inherited diabetes and deafness (MIDD). We confirmed the correlation between diabetes and hearing impairment (*r* ∼ 0.49, *p* < 0.0001), but no regions of linkage were identified (Figure 4, A and B). It is possible that the underlying genetic architecture of these clinical features is more polygenic in structure as is the case in the wider population in the development of hearing impairment^32^ and type II diabetes mellitus.^33^

### Further Characterization of Identified Suggestive Linkage Regions

To assess the merit in pursuing characterization of suggestive linkage regions, we performed additional analysis with the data generated through gene-dropping simulations to establish if the number of suggestive linkage regions identified in each clinical feature was significant (eFigure 3). Linkage analysis identified 6 regions in encephalopathy which surpassed the suggestive of linkage threshold (LOD >2.23). In the analysis of 1,000 simulated data sets, this occurred only once (1/1,000), demonstrating that further investigation into these suggestive regions is merited. In the analysis of stroke-like episodes, 2 suggestive linkage regions were identified; in comparison, 2 or more regions suggestive of genetic linkage were identified in 259/1,000 of the simulated data sets, indicating that although these regions exceed the suggestive linkage threshold for stroke-like episodes (LOD >1.94) they may not denote regions of true genetic linkage.

### Association Analysis

**Figure 5:**
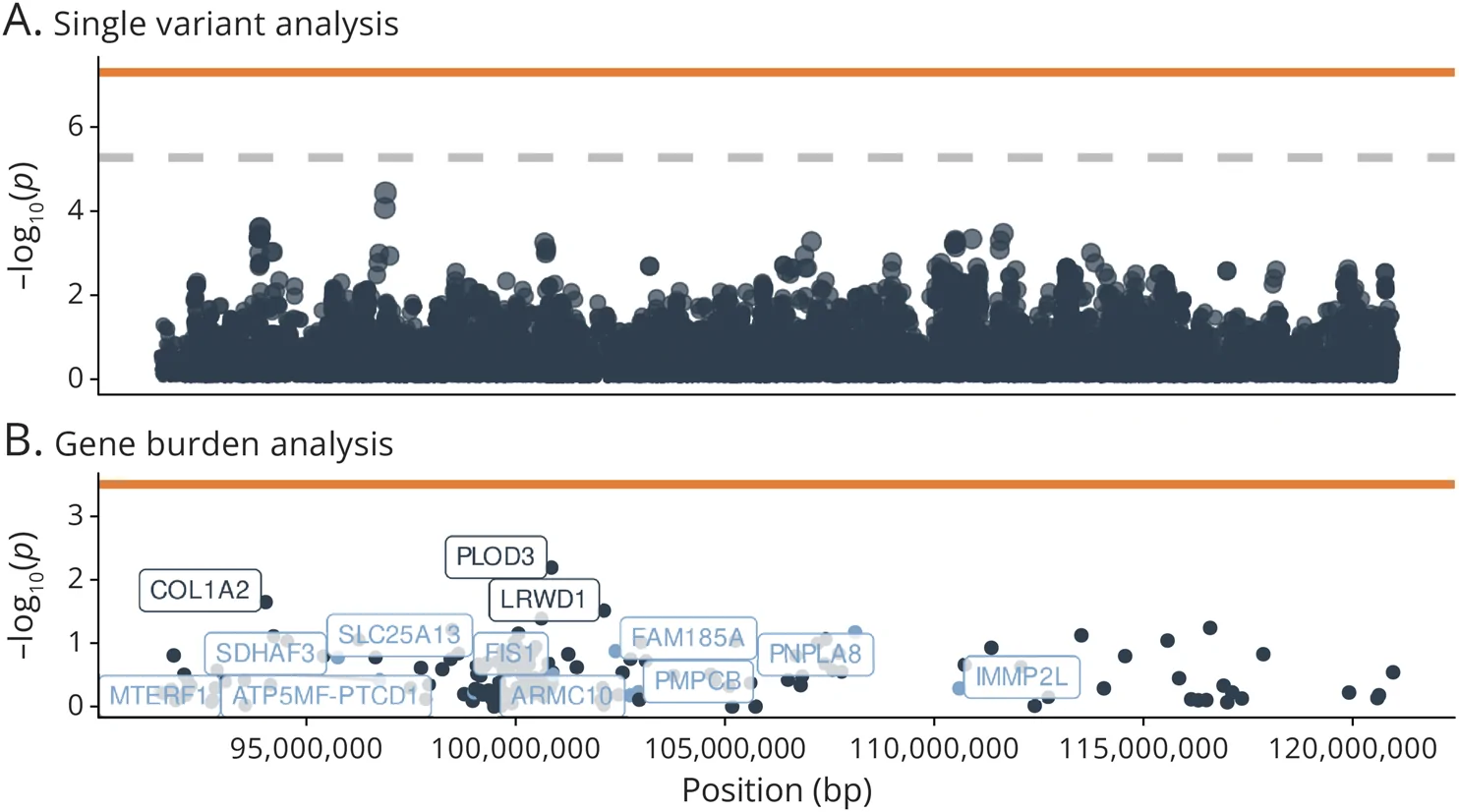
Association Within Chromosome 7 Linkage Peak for Encephalopathy (A). SNP associations with encephalopathy within linkage region on chromosome 7 (54,895 SNPs tested). Orange line represents genome-wide significance level (*p* = 5 × 10^−8^); gray dashed line represents suggestive significance level for the proportion of the genome tested (*p* = 5.3 × 10^−6^); no SNPs passed the suggestive significance threshold; the SNP with the lowest *p* value (*p* = 3.7 × 10^−5^) was 7: 96949935:C:A (rs62500792). (B). Gene burden testing. Genes with a likely mitochondrial function/localization according to MitoCarta3.010 are shown in light blue; orange line represents significance threshold based on Bonferroni correction for 160 genes (*p* = 3.1 × 10^−4^); the gene with the lowest *p* value (*p* = 6.4 × 10^−3^) was PLOD3.

To further investigate the region of chromosome 7 showing significant linkage to encephalopathy, we performed genetic association analysis for 54,895 SNPs (MAF >0.01) across a 30 Mb region spanning the linkage signal. Significance was assessed using a proportional threshold of 5.3 × 10^−6^ based on the variants tested within the region. No significant association signal was detected. However, the SNP with the lowest *p* value (*p* = 3.7 × 10^−5^) was 7:96949935:C:A (rs62500792, Figure 5A; eTable 5). This variant is in strong LD with one other variant (rs62499509; LD > 0.6) and is located in the intergenic region, ∼139 kb downstream of *SDHAF3* and ∼411 kb upstream of *TAC1* (eFigure 4).

Given the potential for genetic heterogeneity to obscure association signals, we used 2 complementary gene-based association approaches. First, we used SAIGE-GENE+^27^ to test exonic variants only, identifying 704 variants mapped to the coding regions of 160 genes within the linkage region (as annotated by ANNOVAR^26^). No gene reached Bonferroni-corrected significance (threshold = 3.1 × 10^−4^), although the lowest *p* value was observed for *PLOD3* (*p* = 6.4 × 10^−3^; Figure 5B, eTable 6).

To broaden the scope beyond coding regions, we then applied MAGMA,^29^ which considers both coding and noncoding variants across all genes within the linkage region. This analysis encompassed 214 genes, reflecting the inclusion of regulatory and intronic variants. Again, no gene surpassed the Bonferroni-corrected threshold (2.3 × 10^−4^), with *IMMP2L* showing the lowest *p* value (*p* = 3.9 × 10^−3^; eTable 7).

## Discussion

Mitochondrial DNA disease is clinically heterogeneous; much of this heterogeneity likely attributable to nuclear genetic variation.^11^ By assembling a unique multicenter collection of m.3243A>G pedigrees and using genetic linkage analysis, we have characterized the nuclear genetic landscape of clinical features associated with MELAS and MIDD, the most common syndromic presentations of m.3243A>G. We demonstrated that encephalopathy and stroke-like episodes, severe neurologic and cardinal features of MELAS, are likely influenced by a small number of nuclear factors with a relatively large effect size. This is supported by the identification of significant genetic linkage to encephalopathy on chromosome 7, and suggestive regions for both encephalopathy (chromosomes 1, 5, 11 and 13) and stroke-like episodes (chromosomes 5 and 6). Both clinical features show linkage to a common region on chromosome 5, which is not surprising given that one is a consequence of the other both acutely and chronically; stroke-like episodes are one cause of acute encephalopathy and recurrent stroke-like episodes cause a chronic encephalopathic state.^34^ By contrast, no linkage regions were identified for diabetes mellitus or hearing impairment, showing that not every clinical feature is influenced by the nuclear genome in the same manner; nuclear factors involved in the development of these features may be more polygenic.

Encephalopathy within m.3243A>G-related disease is highly heritable (h^2^ = 0.64, SE = 0.36, *p* = 0.0408)^11^; the significant linkage region on chromosome 7 is a prime candidate for harboring factor(s) that influence its development. We found no significant association in this region (the top hit was rs62500792; *p* = 3.7 × 10^−5^). The top candidate identified by exonic-variant gene burden testing was *PLOD3* (procollagen-lysine,2-oxoglutarate 5-dioxygenase 3), and for whole-gene burden testing was *IMMP2L* (inner mitochondrial membrane peptidase subunit 2); none of these results were significant after correcting for multiple testing. The lack of association likely reflects limited power to detect small effect sizes, as well as underlying genetic heterogeneity.

Mitochondrial genes within the linkage region are strong candidates. Among these, *MTERF1* is of particular interest. It encodes Mitochondrial Transcription Termination Factor 1 (mTERF1), which binds directly to a sequence within *MT-TL1,* encompassing the 3,243 position. The m.3243A > G variant reduces mTERF binding efficiency, suggesting a plausible mechanistic link.^35,36^ Although neither single-variant nor gene-based association tests reached statistical significance, the limited power of our study means that *MTERF1* cannot be excluded as a candidate.

Heritability (h^2^) estimates suggest a substantial nuclear genetic contribution to the development of m.3243A>G-related hearing impairment.^11^ The absence of linkage regions to both this and diabetes suggests an underlying polygenic architecture, where the nuclear influence is distributed across numerous common variants with small effect sizes, resembling the pattern of genetic risk to non-mitochondrial diabetes mellitus^33^ and hearing loss.^32^ Our results suggest that the manifestation of these clinical feature in m.3243A>G-carriers is likely shaped by a complex interplay of genetic and environmental factors, some of which have been implicated in mitochondrial disorders,^37,38^ as well as shared risk factors present in the general population. Notably, recent findings from a clinically unselected cohort demonstrated that a high type II diabetes genetic risk score increases the penetrance of diabetes among m.3243A>G carriers,^39^ highlighting a convergence between risk factors for both type II and mitochondrial diabetes. Further exploring the overlap between m.3243A>G-related presentations and more prevalent, phenotypically similar traits may yield valuable insights into disease mechanisms and inform more effective genetic counselling strategies.

We acknowledge that there are limitations to our study. Owing to the nature of binary clinical feature information, we have been unable to account for feature severity; it is also important to consider that mildly affected individuals may be mis-classified as unaffected. This challenge is compounded by age-related features, as younger individuals may develop symptoms later in life. Although we included age at assessment as a covariate in all analyses, misclassification could reduce power and contribute to the lack of observed associations. We have primarily used the NMDAS as a guide for binary clinical feature classification, but features are broadly defined, so there is likely to be some heterogeneity within those classed as affected. Furthermore, the NMDAS is a disease assessment tool that can be used in the clinical evaluation of all mitochondrial diseases; therefore, this broader assessment of disease may also miss other manifestations of m.3243A>G-related disease, such as kidney-related disease.^40^ However, using inclusive feature definitions derived from a replicable and clinically validated scale allowed us to combine cohorts; in the research of many rare diseases, there is an important balance to be struck between clinical feature definitions, and statistical power. Finally, individuals referred to the Exeter Genomics Laboratory for diabetes-related genetic testing were not systematically assessed for other clinical features, limiting completeness for some traits.

We have consciously chosen not to correct our genetic linkage results for multiple testing and therefore accept that there is the increased possibility for type I errors. However, considering the rarity of m.3243A>G-related disease, and the unique nature of the cohort used in this work, we maintain that further investigation into these genomic regions using a variety of approaches is justified. The results presented here provide a focus for further investigations, providing guide genomic regions for further exploration, which ultimately will reduce the multiple testing burden moving forward.

Despite assembling a uniquely large cohort of m.3243A>G carriers, our cohort is still relatively small compared with genetic studies for common, complex disease, and we lack a replication cohort. This continues to be an issue when investigating rare disease, presenting challenges to researchers.^41^ However, genetic risk factors with large effect sizes can be detected, as demonstrated for reversible infantile respiratory chain deficiency and digenic Leigh syndrome, both rare mitochondrial diseases caused by variants in both the mitochondrial and nuclear genomes.^42,43^ The challenge of identifying causative variation necessitates the use of a genomic toolbox of strategies such as genetic association tests and whole genome sequencing,^44^ evaluation of polygenic scores associated with related clinical features in the population,^33^ and the use of multidimensional multi-omics data.^45^ It is important that the inability to detect genetic modifiers does not mean that they are not present, indeed, small cohorts combined with genetic heterogeneity have likely hampered past efforts to identify causative nuclear modifiers of mtDNA disease within linkage regions.^46,47^ Our analysis was also mostly restricted to individuals of 1KG-EUR-like genetic ancestry, which limits generalizability. This further emphasizes the value the assembly of larger, international cohorts, and incorporating individuals with diverse genetic ancestry to fully understand mtDNA disease heterogeneity. This work also highlights the importance of the collection of pedigree data, combined with the application and adaptation of methodology previously established for the research of complex disease in the understanding of rare disease.

Understanding disease heterogeneity is a clear priority for mitochondrial disease patients, which is ultimately where research into nuclear modifiers of mtDNA disease will have the biggest, tangible impact.^48^ Our results show that the factors likely to have the biggest impact are those that affect the severe neurologic features associated with MELAS syndrome and have prepared the way for their future identification. This study also demonstrates the importance of assembling large well-phenotyped patient cohorts for rare diseases. A deeper understanding of the genetics and molecular mechanisms underlying m.3243A>G-related clinical features will enable the implementation of personalized medicine and tailored reproductive advice^49,50^ and may identify novel therapeutic targets not only for m.3243A>G-related disease but also for wider mtDNA-related pathologies.

## Glossary

GRM: genetic relationship matrix
LOD: logarithm of the odds
MAF: minor allele frequency
MELAS: mitochondrial encephalomyopathy, lactic acidosis, and stroke-like episodes
MIDD: maternally inherited diabetes and deafness
mtDNA: mitochondrial DNA
NMDAS: Newcastle Mitochondrial Disease Adult Scale
PC: principal component
PCA: principal component analysis
SNP: single nucleotide polymorphism
UCL: University College London
